# Amyloid Precursor Protein A713T Mutation in Calabrian Patients with Alzheimer’s Disease: A Population Genomics Approach to Estimate Inheritance from a Common Ancestor

**DOI:** 10.3390/biomedicines10010020

**Published:** 2021-12-23

**Authors:** Paolo Abondio, Stefania Sarno, Cristina Giuliani, Valentina Laganà, Raffaele Maletta, Livia Bernardi, Francesco Bruno, Rosanna Colao, Gianfranco Puccio, Francesca Frangipane, Barbara Borroni, Christine Van Broeckhoven, Donata Luiselli, Amalia Bruni

**Affiliations:** 1 Laboratory of Molecular Anthropology, Center for Genome Biology, Department of Biological, Geological and Environmental Sciences, University of Bologna, 40126 Bologna, Italy; stefania.sarno2@unibo.it (S.S.); cristina.giuliani2@unibo.it (C.G.); 2 Regional Neurogenetic Center, Azianda Sanitaria Provinciale Catanzaro, 88046 Lamezia Terme, Italy; valelagana@gmail.com (V.L.); raffaelegiovanni.maletta@asp.cz.it (R.M.); liviabernardi@libero.it (L.B.); f.bruno@uniroma1.it (F.B.); colao@arn.it (R.C.); puccio@arn.it (G.P.); francesca.frangipane@libero.it (F.F.); 3 Department of Neurology, University of Brescia, 25121 Brescia, Italy; barbara.borroni@unibs.it; 4 Neurodegenerative Brain Diseases Group, Center for Molecular Neurology, Vlaams Instituut voor Biotechnologie, 2600 Antwerp, Belgium; christine.vanbroeckhoven@uantwerpen.vib.be; 5 Department of Biomedical Sciences, University of Antwerp, 2000 Antwerp, Belgium; 6 Ancient DNA Laboratory, Department of Cultural Heritage, Ravenna Campus, University of Bologna, 48121 Ravenna, Italy; donata.luiselli@unibo.it

**Keywords:** APP, amyloid, Alzheimer’s disease, neurodegeneration, population genomics, common ancestor, genetics, A713T

## Abstract

Mutation A713T in the amyloid precursor protein (APP) has been linked to cases of Alzheimer’s disease (AD), cerebral amyloid angiopathy (CAA) and cerebrovascular disease. Despite its rarity, it has been observed in several families from the same geographical area, in the Calabria region in Southern Italy. Genotyping of 720,000 genome-wide SNPs with the HumanOmniExpress BeadChip was performed for six patients that were representative of apparently unrelated Calabrian families, as well as a Belgian subject of Italian descent (all with the same A713T mutation and disease). Their genomic structure and genetic relationships were analyzed. Demographic reconstruction and coalescent theory were applied to estimate the time of the most recent common ancestor (tMRCA) among patients. Results show that all A713T carriers fell into the genetic variability of Southern Italy and were not more closely related to each other than to any other healthy Calabrian individual. However, five out of seven patients shared a 1.7 Mbp-long DNA segment centered on the A713T mutation, making it possible to estimate a tMRCA for its common origin in the Calabrian region dating back over 1000 years. The analysis of affected individuals with methodologies based on human population genomics thus provides informative insights in support of clinical observations and biomedical research.

## 1. Introduction

Alzheimer’s disease (AD) is the most widespread neurodegenerative disorder, and is primarily characterized by extracellular amyloid plaques, intraneuronal neurofibrillary tangles and synaptic loss [1,2,3]. Genetics plays a key role in the development of AD, with a heritability estimate between 58–79% and over 90% for late-onset and early-onset AD, respectively [4]. Autosomal dominant familial early-onset AD (Alzheimer’s disease, type 1) cases are associated with mutations in the gene encoding for the amyloid precursor protein (APP) located on chromosome 21; indeed, at least twenty-five mutations in the APP gene have been related to AD so far [5]. However, for some APP mutations the inheritance pattern determines whether AD develops. For example, APP_A673T_ mutation causes early-onset AD only in the homozygous state, whereas the heterozygous state protects against AD by decreasing Aβ, Aβ40 and Aβ42 secretion [2,6,7].

Beyond AD, most genetic forms of cerebral amyloid angiopathy (CAA) are also due to mutations in the APP gene [8]. This pathology is primarily characterized by the accumulation of Aβ in cortical and leptomeningeal arteries, arterioles, capillaries and venules [9]. This genetic common ground between AD and CAA raises the question of the co-occurrence of these two diseases. Classical data has shown that mutations in APP located in the Aβ coding region are associated with early-onset AD [10], whereas APP mutations located within or just outside the Aβ coding region are related to CAA [11].

However, some evidence counteracts this usual view. As a first example, a point mutation (Glu693Gln) that flanks the Aβ coding region causes the Dutch-type hereditary CAA (D-CAA; also known as hereditary cerebral hemorrhage with amyloidosis—Dutch type) [11]. This pathology is characterized by early-onset recurrent hemorrhagic strokes and dementia, with few or no dense-core plaques [12,13].

Secondly, an increasing line of evidence suggests a genetic overlap between AD and CAA due to APP mutations located in the Aβ coding region [8,14,15].

Indeed, the APP_A713T_ mutation has consistently been linked to rare cases of familial dominant AD with CAA characterized by several dense-core Aβ plaques and by all the others typical hallmark of AD [16,17,18,19]. APP_A713T_ was initially described as a rare polymorphism or a pathogenic mutation with dominant inheritance and in heterozygosis with incomplete penetrance [16]. The same mutation was later found to cause early- or late-onset dementia with multiple strokes in three members of a family coming from the Calabria Region of Southern Italy. The neuropathological study of the proband revealed the presence of AD with severe CAA and multiple infarcts [17]. Considering these genetic and clinical data, the presence of APP_A713T_ mutation was investigated in 59 patients affected by AD with cerebrovascular lesions (CVLs), a family history of dementia and a neuroradiological evidence of white matter lesions (WMLs) or hypodensities. Data showed a prevalence of 5% for APP_A713T_ mutation. Unexpectedly, patients were three late-onset unrelated individuals living in different areas of the Calabria region and presenting with memory loss, absence of insight, and behavioral and personality changes [18,20]. More recently, the APP_A713T_ mutation was also found in homozygosis in a different and unrelated Calabrian family. These patients were affected by autosomal dominant AD with CVLs and manifested the same clinical phenotype as the heterozygous patients [19]. Taken together, the results of these studies demonstrate that APP_A713T_ mutation is associated with familial AD with early- or late-onset and CVLs due to CAA both in heterozygosis and in homozygosis [16,17,18,19,20], underlining that AD coexists with CAA.

Interestingly, all Italian patients with the APP_A713T_ mutation in heterozygosis [17,18] shared a common DNA haplotype, suggesting that this mutation originated from a putative ancestor, probably living centuries ago in the Calabrian region [20]. In addition, a Belgian AD subject of presumed Italian descent and another AD patient identified in Northern Italy with Calabrian origin were reported, presenting the same APP_A713T_ mutation. Therefore, it appears necessary to carry out a more meticulous study to explore the genomic relationship between reportedly unrelated individuals belonging to a circumscribed population for the presence of such a rare disease.

## 2. Materials and Methods

### 2.1. Dataset Composition

The core dataset of genotyped patients includes seven reportedly unrelated individuals, all affected by AD and carrying the A713T mutation in the amyloid precursor protein (APP): (i) five of these individuals (identified by acronyms PAL, RUF, STR, TAL and PEC) were collected from previously reported families from the Calabrian region of Southern Italy; (ii) one patient was found in Northern Italy, but had a reported Calabrian origin and is identified by the acronym NAP; (iii) another patient, identified by the acronym BEL, was an individual of Italian descent detected in the Walloon region of Belgium, which is historically linked to a massive immigration of Italian coal mine workers (at least 141,000 registered migrants between 1946 and 1958) driven by bilateral economic agreements between Italy and Belgium [21]. For the purposes of the present study, a single AD-patient representative of each of the reported Calabrian families was chosen to minimize the degree of genome sharing among explicitly closely related individuals.

Genotyping of 720K genome-wide SNPs was performed for all seven considered AD patients by using the HumanOmniExpress BeadChip (Illumina, San Diego, CA, USA).

To contextualize the seven AD cases in the genetic variability of Southern Italy, a panel of 84 healthy individuals, already typed for the same set of genome-wide markers, were used as controls [22]. In particular, the control panel included 20 individuals from the province of Benevento as representatives of a non-Calabrian Southern Italian population, as well as 26 and 38 individuals from the Calabrian provinces of Cosenza and Catanzaro, respectively, to approximate the Calabrian area where the affected families presumably originated [22].

Moreover, additional comparisons representative of further areas of Italy were collected from publicly available high-throughput genomic datasets, based on the known patterns of genetic structuring along the Italian peninsula that have been suggested by previous studies based on SNP chip and whole-genome sequencing [23,24]. More precisely, 10 individuals from Bergamo were selected from the Human Genome Diversity Project (HGDP) as a proxy of Northern Italy, 30 individuals from Tuscany were randomly extracted from the 1000 Genome Project (1KGP) as representative of Central Italy, and 24 individuals from the HGDP instead portrayed the more isolated population of Sardinia [25,26].

Finally, a further panel of 30 randomly chosen individuals of Central European ancestry (CEU from the 1KGP) was also added to the genotype-based analysis, to account for the possibility that the Belgian patient could have a higher degree of genome sharing and tighter genetic relationships with individuals from Continental Europe, rather than with (Southern) Italian samples [25].

### 2.2. Quality Controls

In order to assure the quality of the used dataset and remove batch effects, a strict quality control (QC) procedure was applied to genome-wide data using the *PLINK* software version 1.9 (https://www.cog-genomics.org/plink/, accessed on 4 March 2021) [27]. Specifically, only single-nucleotide variants (SNVs) on the autosomal chromosomes were kept for the analyses, and the proportion of missing data per variant (—*geno*) and per individual (—*mind*) was checked by retaining only SNVs and samples showing a missingness proportion lower than 0.05. The respect of Hardy–Weinberg equilibrium (HWE) was also verified for each variant (—*hwe*), by using a threshold of 1.468 × 10^−8^ after Bonferroni correction of multiple testing. Strand flips were checked and resolved as the datasets were merged.

After the QC procedures, the dataset was composed of 185 individuals typed for 509,548 common autosomal SNP markers.

The dataset was further filtered for genotype-based population structure analyses by excluding SNVs with a minor allele frequency lower than 0.01 (—*maf* 0.01), and by using a sliding window of size 50, with a moving step of 5 SNPs and r^2^ threshold of 0.1 (—*indep-pairwise* 50 5 0.1), to remove variants in high linkage disequilibrium (LD). The obtained pruned dataset consisted of 59,980 SNVs.

### 2.3. Genotype-Based Population Structure Analysis

In order to contextualize the ancestry and genomic variability of the analyzed patients and provide a first explorative overview on the genetic relationships between these individuals and the comparison reference populations, a principal component analysis (PCA) based on the pruned genotype data was performed on both the “Italian” local set of 155 (healthy and affected) subjects and by additionally including even the 30 CEU individuals of Central European ancestry. The PCA was executed by using the *convertf* and *smartpca* tools implemented in the *eigensoft* package version 6.0.1 (https://github.com/DReichLab/EIG, accessed on 4 March 2021) [28].

### 2.4. Chromosome Phasing

A haplotype reconstruction procedure was then performed in order to evaluate finer-scale relationship patterns among the seven patients and the Italian control samples. For this purpose, after checking for missing data and for significant deviations from HWE as described in the QC procedure, ambiguous SNVs (carrying an A/T or C/G combination of alleles, for which the chromosome copy and strand could not be univocally defined) were further removed from the unpruned dataset. Then, information about the ancestral or derived nature of each variant was deduced by using a reconstructed reference human genome sequence as a guide for distinguishing between ancestral and derived alleles. In particular, the ancestral/derived state of each allele in such a reference sequence was previously assigned by aligning it with the Ensembl Compara 6 primates EPO genome sequences [29], and only alleles conserved in all the compared genomes were considered as ancestral. Haplotype estimation was finally performed on an ancestral/derived informed dataset of 470,627 SNVs, by using the *SHAPEIT* software version 1.9 (https://mathgen.stats.ox.ac.uk/genetics_software/shapeit/shapeit.html, accessed on 4 March 2021) [30] with default parameter settings and the HapMap phase 3 recombination maps.

### 2.5. Haplotype-Based Analysis

The phased dataset was used to first explore genome-wide haplotype-sharing patterns among individuals by exploiting the approach implemented in CHROMOPAINTER/fineSTRUCTURE [31].

CHROMOPAINTERv2 was used to “paint” the chromosomes of single individuals as a mosaic of genomic segments (or chunks) shared with any other individual in the dataset. In particular, the mutation/emission and the switch rate parameters were first estimated, with 10 steps of the E-M algorithm on a subset of chromosomes {4,10,15,22}. The obtained values were averaged across chromosomes, weighting by number of markers and individuals, and then CHROMOPAINTER was re-run on all chromosomes using the estimated parameters, by considering each individual both as “donor” and as “recipient”.

Subsequently, the obtained matrix of shared “chunk-counts”, combined across the 22 autosomes, was submitted to the fineSTRUCTURE clustering algorithm version fs4.1.1 [31]. fineSTRUCTURE was run by setting 3,000,000 “burn-in” MCMC iterations, followed by 2,000,000 additional iterations where the inferred clustering patterns were sampled every 10,000 runs. Finally, an additional 1,000,000 hill-climbing steps were set to improve posterior probability and obtain the final clustering tree. The obtained population clusters were defined by considering a posterior probability threshold of 90%.

### 2.6. Sharing of Extended Genomic Regions and tMRCA Estimation

The same haplotype phased dataset was then used to specifically explore the sharing of extended portions of genome. The analysis particularly looked at genomic regions on chromosome 21 tagging the APP_A713T_ mutation that are shared among the affected subjects, to ascertain the chance that reportedly unrelated individuals belonging to a circumscribed population would share long regions at the disease-causing mutation by common descent.

The genomic regions encompassing the causal mutation that were found to be shared by the A713T carriers were then used to estimate the time of their most recent common ancestor (tMRCA), by using the analytical approach based on the coalescent theory as described in Pagani and colleagues [32].

## 3. Results

To preliminarily characterize the variability of analyzed patients in the context of the broader Italian genetic diversity, a principal component analysis (PCA) was performed including data from healthy comparison populations as control groups. As expected, the distribution of genomic variation in Italy appears to be tri-partitioned, with a clear differentiation of Sardinians from continental Italy, and a different clustering pattern between North-Central and Southern peninsular Italians, respectively [23]. In this context, all seven A713T carriers (including the Belgian subject) were revealed to fall neatly within the genetic variability of the Southern Italian population, thus suggesting their localized geographical origin in the Calabrian region (Supplementary Figure S1a). Importantly, the same pattern was confirmed even when further comparisons from Central Europe (CEU) were additionally included in the PCA plot (Supplementary Figure S1b).

**Figure 1:**
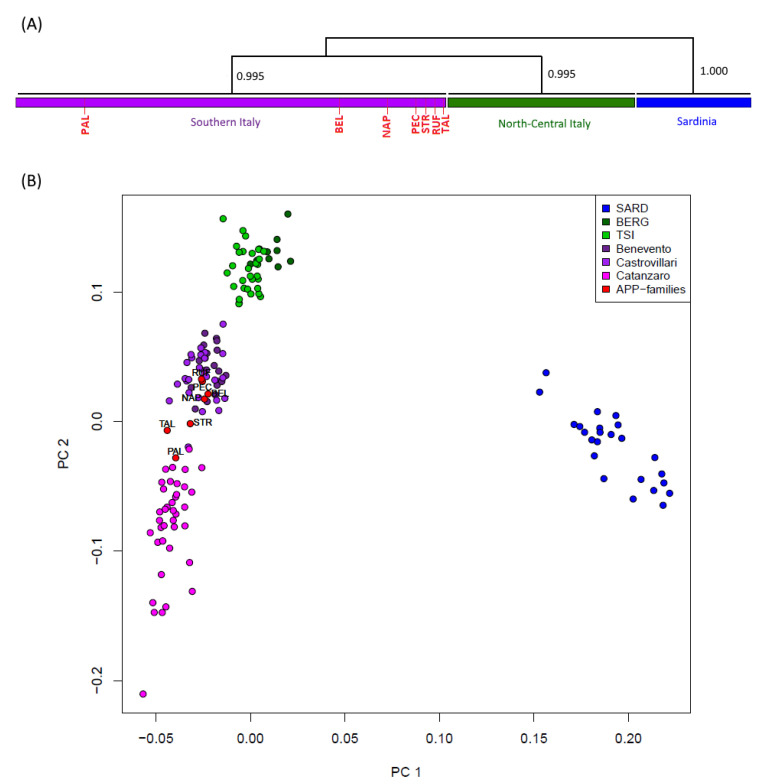
Genetic structure based on genome-wide haplotype-sharing pattern. (**A**) Schematic representation of fineSTRUCTURE hierarchical clustering showing the relationships among analyzed patients and the Italian healthy controls. Population clusters were defined by considering a posterior probability threshold higher than 90%. The full fineSTRUCTURE tree is shown in Supplementary Figure S3. (**B**) Principal component analysis based on chunkcount coancestry matrix, obtained from CHROMOPAINTER profiles; the top two components have been plotted in a scatterplot.

The CHROMOPAINTER/fineSTRUCTURE pipeline was then applied to phased data to evaluate genomic relationships among Italian individuals (including both healthy and affected subjects) based on genome-wide haplotype-sharing patterns. The CHROMOPAINTER algorithm indeed provides estimates of everyone’s DNA as the combination of genomic chunks shared with the other members of the dataset, and the obtained coancestry matrix is then used to describe clusters of genetically homogeneous individuals (Supplementary Figures S2 and S3). The dendrogram drawn from the genomic painting profiles confirms the substantial evidence, which also emerged from genotype-based analyses, of a common genetic affinity between all the patients and the people of Calabrian descent. Accordingly, all seven A713T carriers clearly distributed into the Southern Italian clade, as further confirmed by the same PCA performed on the obtained coancestry matrix (Figure 1).

Overall, both genotype-based and haplotype-based analyses thus supported the Calabrian origin of all seven patients. Therefore, the extent of relatedness at the genome-wide level was assessed among the Southern Italian samples, estimating the total proportion of genome shared by descent (IBD) among pairs of individuals (using the —*genome* PLINK function). In particular, the extent of genome-wide sharing between pairs of patients was compared to that of pairs of affected and control samples. The whole proportion of genome shared IBD by the pairs of the seven A713T carriers, each belonging to a different family, was comparable to the proportion of genome shared IBD between these patients and the healthy Southern Italian controls (i.e., no patient–patient comparison was above the top 1% of the empirical distribution of patient–control comparisons). This confirms that, at a genomic level, the patient families were not closely related to each other.

**Figure 2:**
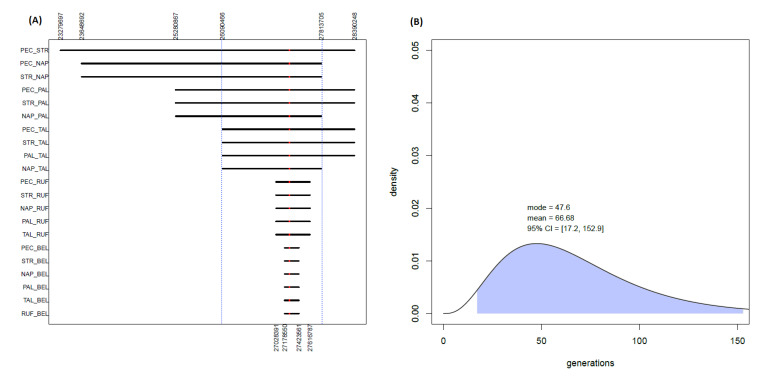
Sharing of extended genomic regions and tMRCA estimation. (**A**) Observed genomic regions tagging the APPA713T mutation shared among pairs of patients. The blue vertical bars highlight the genomic region of 300 SNPs and 1.7 Mbp shared among five of the seven patients. (**B**) Estimated tMRCA based on the highlighted genomic region.

However, the worldwide rarity of the AD-causing A713T mutation challenges the findings of such a high number of apparently unrelated affected families in a restricted and circumscribed population, raising the question that they may have shared a common ancestor many generations ago. Thanks to an ad hoc Python script, pairwise comparisons between the seven AD-affected subjects were performed at a local genomic level to assess the sharing of extended portions of genetic material around the causative mutation on chromosome 21 (Figure 2A). Indeed, long common tracts shared among the patients were found. In particular, the A713T mutation was found to be included in a genomic region (spanning 300 SNPs and 1.72 Mbp) which is shared among five out of the seven analyzed patients (PEC, STR, PAL, NAP and TAL). Furthermore, smaller portions of the same region are also shared with the other two remaining A713T carriers: namely, 110 SNPs and 0.59 Mbp with RUF, and 47 SNPs and 0.25 Mbp with BEL, respectively.

In order to statistically validate the results, the probability was checked that the same sets of patients would share, along the rest of the genome, other tracts with the same extension (in terms of either number of SNPs, RankSNPs, or bp dimension, RankDIM(bP)) of the found A713T-shared regions. In particular, the possibility of sharing by chance such long segments was evaluated both between the five patients sharing the longer region of 300 SNPs and 1.72 Mbp, as well as between the six and all seven patients instead sharing shorter tracts of it (Supplementary Figure S4). In all cases, the probability of observing other genomic regions shared among the considered patients that are at least as large as the one including the A713T mutation was lower than 0.05 (RankSNPs = 2.61 × 10^−5^ and RankDIM(bP) = 1.83 × 10^−4^ for five patients; RankSNPs = 2.96 × 10^−5^ and RankDIM(bP) = 3.56 × 10^−4^ for six patients; RankSNPs = 1.01 × 10^−4^ and RankDIM(bP) = 1.41 × 10^−3^ for seven patients). More precisely, no other region shared by the same patients and with a higher number of SNPs than the one centered on the A713T mutation was detected throughout the rest of the genome. Only in the case of the shortest window shared among all seven patients were two other regions observed with an analogous (i.e., 47) or slightly higher (i.e., 51) number of SNPs (Supplementary Figure S4). Furthermore, in terms of length in bp, any other shared region in the genome showing higher dimensions than the A713T-shared region was however generally characterized by a significant much lower number of SNPs (Supplementary Figure S4).

In parallel, we evaluated the probability that each of the analyzed patients would share with the Southern Italian controls a region of the same dimension (in terms of either number of SNPs, PSNPs or bp length, PDIM(bP)) of the A713T region instead shared with the other patients. To do this, the genome of each member of the group of five, six or seven patients was sequentially compared to that of all the Southern Italian controls and checked for windows of the specified bp length or containing a corresponding number of SNPs. We determined that a segment of at least 300 SNPs as the one shared by the five patients is unlikely to be shared along the rest of the genome between any patient and the Calabrian controls, even after Bonferroni correction for multiple testing, providing a threshold *p*-value of 6 × 10^−5^ (Supplementary Table S1). However, the same does not apply for the smaller segments shared by the six or all seven patients, for which it was possible to find along the rest of the genome segments of analogous (or higher) dimensions shared by the patients with the controls (Supplementary Table S1).

Therefore, in the case of these shorter regions it could be not excluded that people of Southern Italy would share with the patients segments of the same dimension of the one instead including the AD-causing A713T mutation. On the other hand, the length of the longer segment encompassing the APP_A713T_ mutation and shared among at least five of the seven patients would make it unlikely to be shared by chance, instead suggesting its possible sharing from a common descent. Indeed, the identification of an extended genomic segment, centered on the causative A713T mutation and shared among the considered AD cases, allowed us to estimate the time of the most recent common ancestor (tMRCA) of that genomic region, using the analytic approach based on coalescent theory reported in Pagani and colleagues [32]. For the longer genomic region of 300 SNPs and 1.7 Mbp, which is shared among five of the seven patients, this provided a mean estimate of 66.6 generations ago (with the mode being 47.6 generations) for the time of their most recent common ancestor (Figure 2B). On the other hand, the smaller regions shared with also the sixth and with the seventh patient produced larger estimates (Supplementary Figure S5) that are respectively more than four and more than eight times higher than the one observed for the other five patients, pushing the tMRCA back to hundreds of generations ago.

## 4. Discussion

The present study was performed to explore the genomic relationships between seven reportedly unrelated individuals affected by AD with CVLs due to CAA, carrying the same APP_A713T_ mutation and belonging to a circumscribed population in the Calabrian region of Southern Italy. The mutation is exceedingly rare in the worldwide population (with only one heterozygous carrier in 2504 apparently healthy subjects from the 1000 Genomes Project (1KGP), but has been repeatedly associated to cases of AD with CVLs due to CAA both in isolated individuals and in specific Italian families over several generations [17,18,19,20]. As described at the end of this section, by considering the genetic makeup and the genomic relationships of affected individuals carrying peculiar mutations, the analysis of these cases with methodologies based on human population genomics could provide informative insights in support of clinical observations and biomedical research.

At a broadest genetic level, the results presented in this study clearly support a Southern Italian origin for all seven AD cases here considered, including the ones found in Belgium and Northern Italy, even when representatives of a population of Central European ancestry were included in the analysis. A common genetic affinity is highlighted between the patients and the people of Calabrian descent, that indeed cluster together by forming a separate group from the representatives of Northern/Central Italy, with all seven APP_A713T_ carriers falling neatly into the genetic diversity of the Southern Italian clade (Figure 1, Supplementary Figure S2).

Interestingly, the estimated total proportion of genome shared among pairs of affected individuals is comparable to that between pairs of patients and healthy Southern Italian controls, showing that, at a genome-wide level, the patients’ families are not closely related to each other. However, the analyses performed on the APP_A713T_ carriers highlight a statistically significant degree of genetic sharing in a circumscribed genomic region surrounding the specific mutation, which is absent for the other Southern Italian controls. Given the worldwide rarity of such an event, the sharing of long genomic tracts at the disease-causing locus suggests the possibility that the mutated SNP may have been inherited by a common ancestor who lived many generations ago, instead of the mutation having appeared independently in several distinct pedigrees from the same geographic area. Accordingly, a segment as long as the one shared by the APP_A713T_ carriers is unlikely to be shared along the rest of the genome between any patient and the Calabrian controls. The segment carrying 300 SNPs and the 1.7 Mbp-long APP_A713T_ shared among five of the seven cases (including the Northern Italian sample of Southern Italian origin) indeed allowed for the computation of a tMRCA with a mean of 66.7 generations ago and mode of 47.6 generations ago, which is consistent with previous estimates for a common origin of the APP_A713T_ mutation in the Calabrian region dating back over 1000 years ago [18,20].

A shorter portion of the same genomic tract was also detected among the other APP_A713T_ carriers, including the Belgian AD-affected individual, providing the interesting opportunity to explore the possibility that this APP_A713T_ mutation indeed has an Italian origin and is linked to a “founder event”. The Belgian subject shares with the other six cases a reduced portion of the genome (47 SNPs and 0.25 Mbp) surrounding the putatively causative mutation, which puts the estimate for the tMRCA further back in time, to a period of hundreds of generations ago in which virtually all individuals would have shared a common ancestor. While in this case it could not be excluded that people of Southern Italy would share with the patient other segments of the same dimension, the rarity of the mutation tagging the region instead shared by the patients would make it unlikely that they share it by chance. One possibility is that a more recent recombination event effectively split the 300 SNP-long segment, so that the Belgian individual inherited only a 47 SNP-long portion including the putatively causative variant.

From a clinical standpoint, considering the pathogenicity of APP_A713T_ mutation both in heterozygous and homozygous states [17,18,19,20] and the high rates of migrations from Calabria region to the rest of the world over many centuries [33], the results presented here suggest that APP_A713T_ analysis should be considered for the genetic screening of early- or late-onset AD patients, especially when manifesting CVLs due to CAA and with a family history of dementia. This study provides new insights about the genomic relationships of affected individuals carrying APP_A713T_ mutation and, thanks to the analysis of a wide genomic region including the causative variant, crucial information can be gained about the origin and spreading of this rare pathological mutation.

## Data Availability

The data used in this study will be made available upon reasonable request addressed to the corresponding authors of this paper.

## Supplementary Materials

The following are available online at https://www.mdpi.com/article/10.3390/biomedicines10010020/s1. Figure S1: Principal component analysis based on genotype-based data. Figure S2: Heatmap of the CHROMOPAINTER coancestry matrix. Figure S3: Full dendrogram of the fineSTRUCTURE hierarchical clustering analysis. Figure S4: Scatterplot of the number of SNPs vs. bp size of observed regions along the genome shared among patients. Figure S5: tMRCA estimation for small windows. Table S1: Window size and SNP number probability.
