# Supplementary material for: Amyloid Precursor Protein A713T Mutation in Calabrian Patients with Alzheimer’s Disease: A Population Genomics Approach to Estimate Inheritance from a Common Ancestor
Source: Biomedicines. 2021 Dec 23;10(1):20. doi: 10.3390/biomedicines10010020 (PMC8773445; doi:10.3390/biomedicines10010020)
Supplement: Supplementary file 1 [file biomedicines-10-00020-s001.zip › biomedicines-1495596-supplementary.pdf]

Supplementary figures

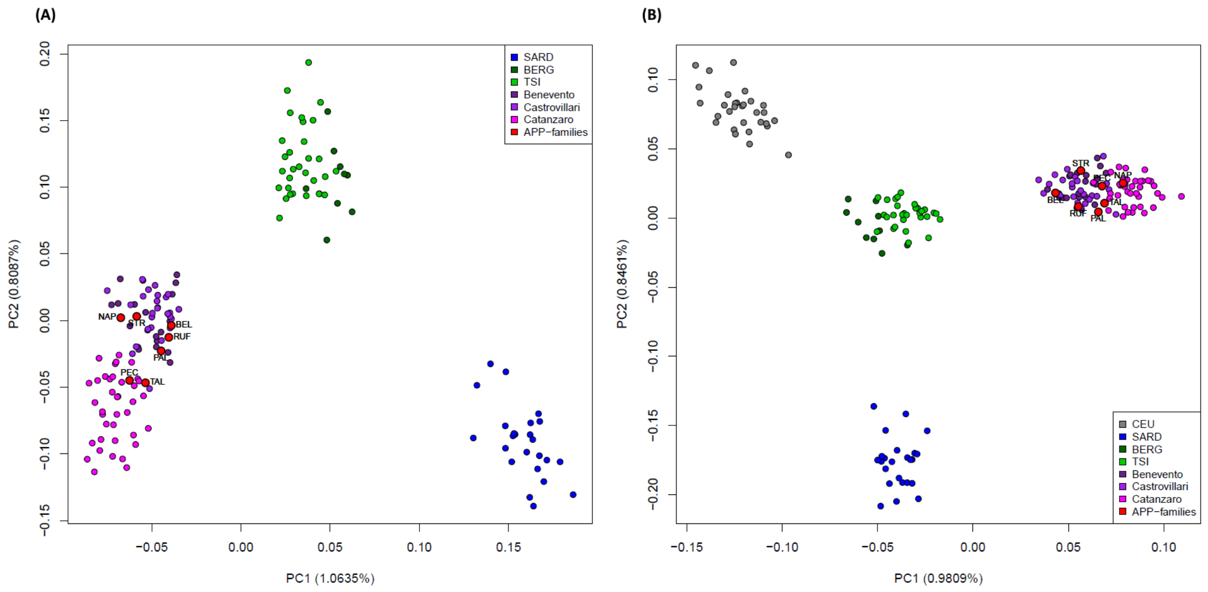

Supplementary Figure S1. Principal component analysis based on genotype-based data. Scatterplots of the first vs. second PCs are reported for both (A) the PCA performed on the “Italian” local set of 155 healthy and affected subjects as well as (B) by additionally including further comparisons from Central Europe. CEU: Central European Ancestry; SARD: Sardinia; BERG: Bergamo; TSI: Individuals from Tuscany, Italy.

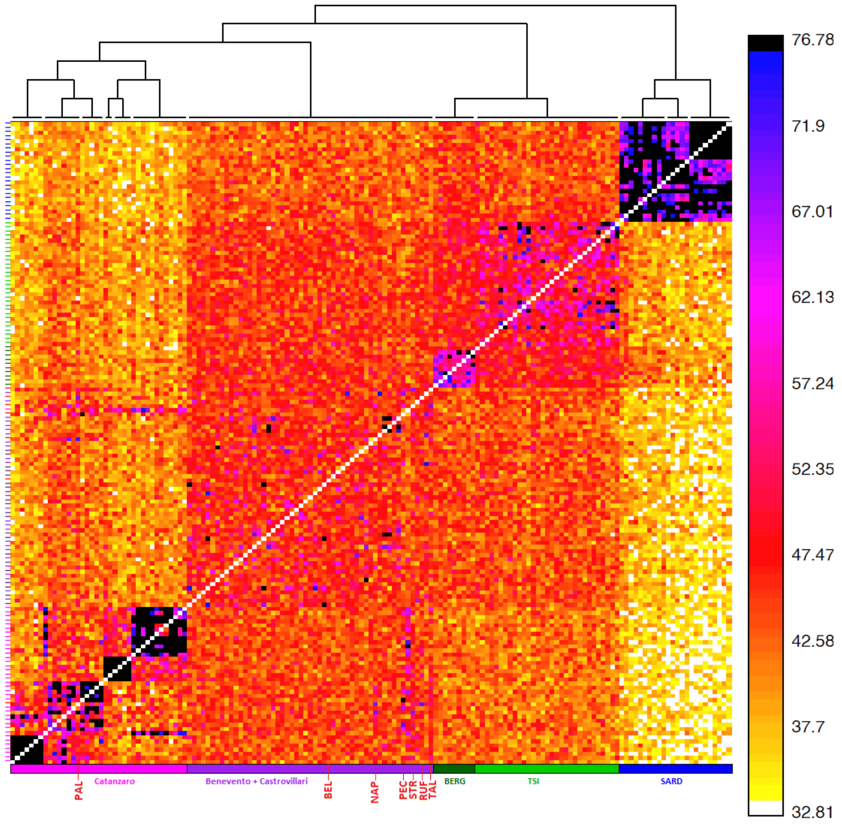

Supplementary Figure S2. **Heatmap of the CHROMOPAINTER coancestry matrix.** It reports the total length of haplotypes shared between the pairs of individuals.

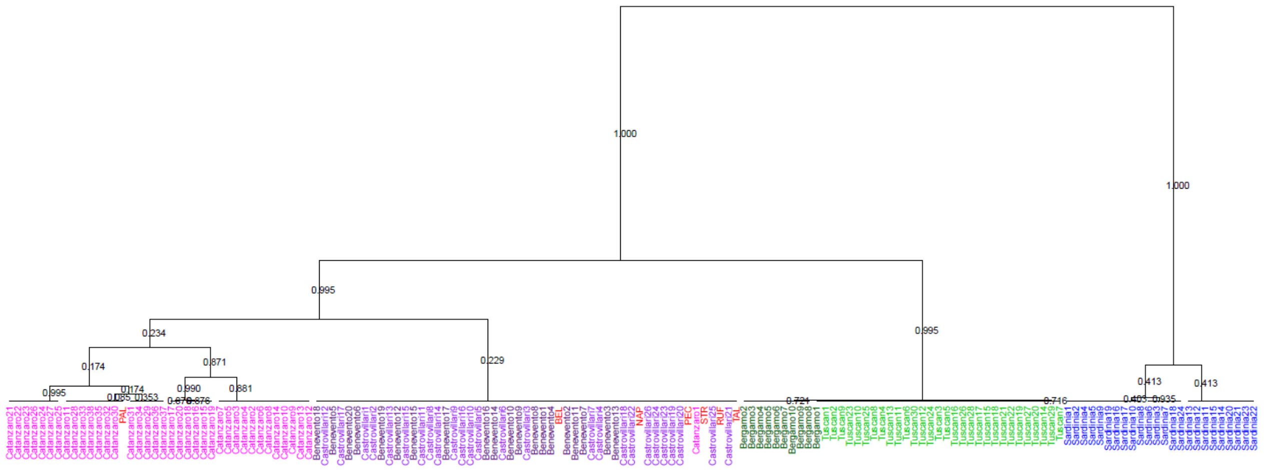

Supplementary Figure S3. **Full dendrogram of the fineSTRUCTURE hierarchical clustering analysis.** The labels detail the population name of each Italian healthy subject and the acronym of the analyzed patients. For each branch, the obtained posterior probability is reported.

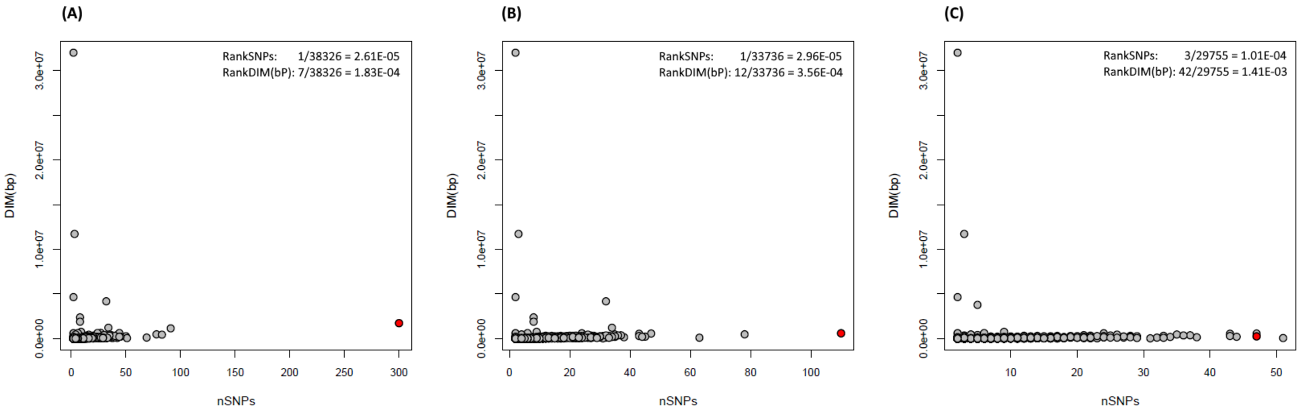

Supplementary Figure S4: **Scatterplot of the number of SNPs vs. bp size of observed regions along the genome shared among patients.** For (A) five; (B) six; (C) seven cases are reported the probability ($Rank_{SNP}$, $Rank_{DIM(bP)}$) of observing windows with higher dimensions with respect to the one including the APP_{A713T} mutation (in red).

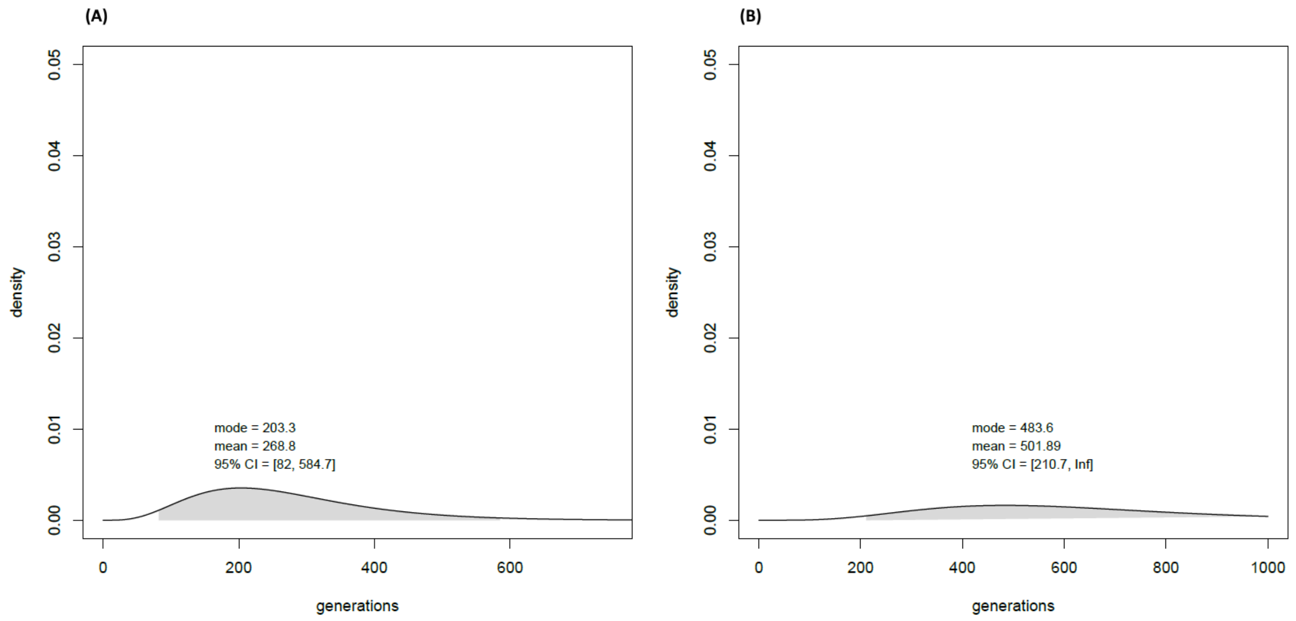

Supplementary Figure S5: **tMRCA estimation for small windows**. Estimated tMRCA based on (A) the window of 110 SNPs and 0.59 Mbp shared among six patients; (B) the window of 47 SNPs and 0.25 Mbp shared among all seven patients.

Supplementary tables

Supplementary Table S1: Window size and SNP number probability. Probability of observing a window of bigger size (pDIM(bp)) or with a higher number of SNPs (pSNPs) shared between each patient and all the Southern Italian healthy controls, with respect to the segment shared among five, six or seven patients respectively. Threshold of significance is 6.0E-05 after Bonferroni correction.

	**nWID**	**$Higher_{SNPs}$**	**$Higher_{DIM(bp)}$**	**p_{SNPs}**	**$p_{DIM(bp)}$**
**Segment: 300 SNPs and 1.723.239 bp**					
NAP	7906408	141	2746	1.78E-05	3.47E-04
PAL	7927467	147	2537	1.85E-05	3.20E-04
PEC	7899337	92	2191	1.16E-05	2.77E-04
STR	7894593	163	2549	2.06E-05	3.23E-04
TAL	7919537	88	2413	1.11E-05	3.05E-04
**Segment: 110 SNPs and 588.396 bp**					
NAP	7906408	4955	20634	6.27E-04	0.00261
PAL	7927467	4991	21294	6.30E-04	0.00269
PEC	7899337	4786	21115	6.06E-04	0.00267
RUF	7910828	4788	20116	6.05E-04	0.00254
STR	7894593	5243	20705	6.64E-04	0.00262
TAL	7919537	5028	20101	6.35E-04	0.00254
**Segment: 47 SNPs and 245.011 bp**					
BEL	7921910	87126	136527	0.01100	0.01723
NAP	7906408	87952	136122	0.01112	0.01722
PAL	7927467	88116	137572	0.01112	0.01735
PEC	7899337	88983	136607	0.01126	0.01729
RUF	7910828	88006	136848	0.01112	0.01730
STR	7894593	90341	138624	0.01144	0.01756
TAL	7919537	87461	136165	0.01104	0.01719
